# Supplementary material for: Molecular mechanism of choline and ethanolamine transport in humans
Source: Nature. 2024 May 22;630(8016):501–8. doi: 10.1038/s41586-024-07444-7 (PMC11168923; doi:10.1038/s41586-024-07444-7)
Supplement: Supplementary file 1 — Supplementary Tables 1–4 and Figs. 1–12. [file 41586_2024_7444_MOESM1_ESM.pdf]

nature portfolio

https://doi.org/10.1038/s41586-024-07444-7

Supplementary information

Molecular mechanism of choline and ethanolamine transport in humans

In the format provided by the authors and unedited

Molecular mechanism of choline and ethanolamine transport in humans

Keiken Ri[1-5†], Tsai-Hsuan Weng[6†], Ainara Claveras Cabezudo[7,8†], Wiebke Jösting[6], Yu Zhang[1], Andre Bazzone[9], Nancy C.P. Leong[1-5], Sonja Welsch[10], Raymond T. Doty[11], Gonca Gursu[6], Tiffany Jia Ying Lim[1-5], Sarah Luise Schmidt[6], Janis L. Abkowitz[11], Gerhard Hummer[7,12*], Di Wu[6,15*], Long N Nguyen[1-5*], Schara Safarian[6,13-15*]

Affiliations

[1] Department of Biochemistry, Yong Loo Lin School of Medicine, National University of Singapore, Singapore 119228

[2] Immunology Program, Life Sciences Institute, National University of Singapore, Singapore 117456

[3] Singapore Lipidomics Incubator (SLING), Life Sciences Institute, National University of Singapore, Singapore 117456

[4] Cardiovascular Disease Research (CVD) Programme, Yong Loo Lin School of Medicine, National University of Singapore, Singapore 117545

[5] Immunology Translational Research Program, Yong Loo Lin School of Medicine, National University of Singapore, Singapore 117456

[6] Department and Emeritus Group of Molecular Membrane Biology, Max Planck Institute of Biophysics, D-60438 Frankfurt/Main, Germany

[7] Department of Theoretical Biophysics, Max Planck Institute of Biophysics, D-60438 Frankfurt/Main, Germany

[8] IMPRS on Cellular Biophysics, D-60438 Frankfurt/Main, Germany

[9] Nanion Technologies GmbH, Ganghoferstr. 70a, 80339, Munich, Germany

[10] Central Electron Microscopy Facility, Max Planck Institute of Biophysics, D-60438 Frankfurt am Main, Germany

[11] Division of Hematology, Department of Medicine, University of Washington, Seattle, WA, 98195, USA

[12] Institute of Biophysics, Goethe University Frankfurt, D-60438 Frankfurt/Main, Germany

[13] Institute of Clinical Pharmacology, Goethe University Frankfurt, D-60438 Frankfurt/Main, Germany

[14] Fraunhofer Cluster of Excellence for Immune Mediated Diseases CIMD, D-60438 Frankfurt/Main, Germany

[15] Fraunhofer Institute for Translational Medicine and Pharmacology ITMP Frankfurt, D-60438 Frankfurt/Main, Germany

*Correspondence and requests for materials should be addressed to Gerhard Hummer (gerhard.hummer@biophys.mpg.de), Di Wu (di.wu@biophys.mpg.de), Long Nguyen (bchnnl@nus.edu.sg), and Schara Safarian (schara.safarian@biophys.mpg.de).

†These authors contributed equally.

This file includes:

Supplementary Tables 1–4

Supplementary Figures 1–12

Other Supplementary Material for this manuscript includes the following:

Supplementary Videos 1–4

Supplementary Table 1: Nucleotide sequences of human FLVCR variants used in this study.

Variant	Nucleotide sequence
FLVCR1 (wild-type)	gacggatcgggagatctcccgatcccctatggtgcactctcagtacaatctgctctgatgccgcatagttaagccagtatctgctccctgcttgtgtgttggaggtcgctgagtagtgcgcgagcaaaatttaagctacaacaaggcaaggcttgaccgacaattgcatgaagaatctgcttagggttaggcgttttgcgctgcttcgcgatgtacgggccagatatacgcgttgacattgattattgactagttattaatagtaatcaattacggggtcattagttcatagcccatatatggagttccgcgttacataacttacggtaaatggcccgcctggctgaccgcccaacgacccccgcccattgacgtcaataatgacgtatgttcccatagtaacgccaatagggactttccattgacgtcaatgggtggagtatttacggtaaactgcccacttggcagtacatcaagtgtatcatatgccaagtacgccccctattgacgtcaatgacggtaaatggcccgcctggcattatgcccagtacatgaccttatgggactttcctacttggcagtacatctacgtattagtcatcgctattaccatggtgatgcggttttggcagtacatcaatgggcgtggatagcggtttgactcacggggatttccaagtctccaccccattgacgtcaatgggagtttgttttggcaccaaaatcaacgggactttccaaaatgtcgtaacaactccgccccattgacgcaaatgggcggtaggcgtgtacggtgggaggtctatataagcagagctctccctatcagtgatagagatctccctatcagtgatagagatcgtcgacgagctcgtttagtgaaccgtcagatcgcctggagacgccatccacgctgttttgacctccatagaagacaccgggaccgatccagcctccggactctagcgtttaaacttaagcttgccaccatggcgcggccagacgatgaggagggggcggcggtggcgcccggacacccgctcgcgaaaggatacctcccgttgccgaggggcgcgcccgttgggaaggagagcgtggagctgcagaacgggcccaaagcgggcaccttcccggtgaatggggcccccgggacagcctcgctgccgcctcgggagttctgggcggcctcagactccactggccccagaagaggagacccaggcccggctgctgcctgcgggcgcgggagctgagaccccggggccgagagcagcccgctgccccttacggcgctctccccgcggcgcttcgtggtgctcctgatcttcagcctgtactcgctggtcaacgcctttcagtggatccagtacagcatcattagcaacgtcttcgagggcttctacggtgtcaccttgctgcacatcgactggctgtccatggtgtacatgctggcctacgtgcccctcatcttcccggccacctggctgctggacaccagaggcctgcggctcaccgccctgctgggctccggcctcaactgcctgggtgcctggatcaagtgcggcagtgtgcagcagcatctcttctgggtcaccatgttgggccagtgcttgtgctcggtggcccaggtgttcatcctgggcttgccctcccgcatcgcctcagtgtggtttgggcccaaagaggtgtccacagcttgtgccaccgccgtgctgggcaatcagcttggaactgcagttggctttttgctaccaccagttttagtacccaacacacagaatgacacaaatctcctggcttgtaatatcagcaccatgttttatggaacatcagctgttgccacacttttatttattttaacagcaattgccttcaaagaaaaacctcggtatccaccaagtcaggctcaagcagctcttcaagacagtcccctgaagagtactcctataagaaatcaataagaaacctgtttaaaaacattccttttgtccttctgttgatcacttatggtatcatgactggtgccttttattcagtctcaacgttattaaatcaaatgatattgacatattatgagggagaagaagtcaatgctggaaggattgggctaacgctagtagtagctggaatggtgggctctattctttgtggcttatggctggattatactaaaacatacaaacagactactctgatagtttatattttgtcttttattggaatggttatctttactttcacattggaccttagatatattatcatcgtgtttgttactggaggggtgcttggcttcttcatgactggttacctccctttgggttttgaatttgctgttgaaatcacttaccctgaatctgaaggtacttcatctggtcttcttaatgcttctgcacagatatttggaattttgttcacattggctcaaggaaagctcacatcagactatggtcctaaggcagggaacatttttctctgtgtctggatgtttataggcatcatattaacagcattaatcaagtctgatctgcgaagacacaacataaatataggaattacaaatgttgatgttaaagctataccagctgacagtcccacagaccaagaaccaaaaacggttatgttgtccaagcagtcagaatcagcaattgattacaaggatgacgacgataagtgagcggccgctcgagtctagagggcccgtttaaacccgctgatcagcctcgactgtgccttctagttgccagccatctgttgtttgcccctcccccgtgccttccttgaccctggaaggtgccactcccactgtcctttcctaataaaatgaggaaattgcatcgcattgtctgagtaggtgtcattctattctggggggtggggtggggcaggacagcaagggggaggattgggaagacaatagcaggcatgctggggatgcggtgggctctatggcttctgaggcggaaagaaccagctggggctctagggggtatccccacgcgccctgtagcggcgcattaagcgcggcgggtgtggtggttacgcgcagcgtgaccgctacacttgccagcgccctagcgcccgctcctttcgctttcttcccttcctttctcgccacgttcgccggctttccccgtcaagctctaaatcgggggctccctttagggttccgatttagtgctttacggcacctcgaccccaaaaaacttgattagggtgatggttcacgtacctagaagttcctattccgaagttcctattctctagaaagtataggaacttccttggccaaaaagcctgaactcaccgcgacgtctgtcgagaagtttctgatcgaaaagttcgacagcgtctccgacctgatgcagctctcggagggcgaagaatctcgtgctttcagcttcgatgtaggagggcgtggatatgtcctgcgggtaaatagctgcgccgatggtttctacaaagatcgttatgtttatcggcactttgcatcggccgcgctcccgattccggaagtgcttgacattggggaattcagcgagagcctgacctattgcatctcccgccgtgcacagggtgtcacgttgcaagacctgcctgaaaccgaactgcccgctgttctgcagccggtcgcggaggccatggatgcgatcgctgcggccgatcttagccagacgagcgggttcggcccattcggaccgcaaggaatcggtcaatacactacatggcgtgatttcatatgcgcgattgctgatccccatgtgtatcactggcaaactgtgatggacgacaccgtcagtgcgtccgtcgcgcaggctctcgatgagctgatgctttgggccgaggactgccccgaagtccggcacctcgtgcacgcggatttcggctccaacaatgtcctgacggacaatggccgcataacagcggtcattgactggagcgaggcgatgttcggggattcccaatacgaggtcgccaacatcttcttctggaggccgtggttggcttgtatggagcagcagacgcgctacttcgagcggaggcatccggagcttgcaggatcgccgcggctccgggcgtatatgctccgcattggtcttgaccaactctatcagagcttggttgacggcaatttcgatgatgcagcttgggcgcagggtcgatgcgacgcaatcgtccgatccggagccgggactgtcgggcgtacacaaatcgcccgcagaagcgcggccgtctggaccgatggctgtgtagaagtactcgccgatagtggaaaccgacgccccagcactcgtccgagggcaaaggaatagcacgtactacgagatttcgattccaccgccgccttctatgaaaggttgggcttcggaatcgttttccgggacgccggctggatgatcctccagcgcggggatctcatgctggagttcttcgcccaccccaacttgtttattgcagcttataatggttacaaataaagcaatagcatcacaaatttcacaaataaagcatttttttcactgcattctagttgtggtttgtccaaactcatcaatgtatcttatcatgtctgtataccgtcgacctctagctagagcttggcgtaatcatggtcatagctgtttcctgtgtgaaattgttatccgctcacaattccacacaacatacgagccggaagcataaagtgtaaagcctggggtgcctaatgagtgagctaactcacattaattgcgttgcgctcactgcccgctttccagtcgggaaacctgtcgtgccagctgcattaatgaatcggccaacgcgcggggagaggcggtttgcgtattgggcgctcttccgcttcctcgctcactgactcgctgcgctcggtcgttcggctgcggcgagcggtatcagctcactcaaaggcggtaatacggttatccacagaatcaggggataacgcaggaaagaacatgtgagcaaaaggccagcaaaaggccaggaaccgtaaaaaggccgcgttgctggcgtttttccataggctccgcccccctgacgagcatcacaaaaatcgacgctcaagtcagaggtggcgaaacccgacaggactataaagataccaggcgtttccccctggaagctccctcgtgcgctctcctgttccgaccctgccgcttaccggatacctgtccgcctttctcccttcgggaagcgtggcgctttctcatagctcacgctgtaggtatctcagttcggtgtaggtcgttcgctccaagctgggctgtgtgcacgaaccccccgttcagcccgaccgctgcgccttatccggtaactatcgtcttgagtccaacccggtaagacacgacttatcgccactggcagcagccactggtaacaggattagcagagcgaggtatgtaggcggtgctacagagttcttgaagtggtggcctaactacggctacactagaagaacagtatttggtatctgcgctctgctgaagccagttaccttcggaaaaagagttggtagctcttgatccggcaaacaaaccaccgctggtagcggtggtttttttgtttgcaagcagcagattacgcgcagaaaaaaaggatctcaagaagatcctttgatcttttctacggggtctgacgctcagtggaacgaaaactcacgttaagggattttggtcatgagattatcaaaaaggatcttcacctagatccttttaaattaaaaatgaagttttaaatcaatctaaagtatatatgagtaaacttggtctgacagttaccaatgcttaatcagtgaggcacctatctcagcgatctgtctatttcgttcatccatagttgcctgactccccgtcgtgtagataactacgatacgggagggcttaccatctggccccagtgctgcaatgataccgcgagacccacgctcaccggctccagatttatcagcaataaaccagccagccggaagggccgagcgcagaagtggtcctgcaactttatccgcctccatccagtctattaattgttgccgggaagctagagtaagtagttcgccagttaatagtttgcgcaacgttgttgccattgctacaggcatcgtggtgtcacgctcgtcgtttggtatggcttcattcagctccggttcccaacgatcaaggcgagttacatgatcccccatgttgtgcaaaaaagcggttagctccttcggtcctccgatcgttgtcagaagtaagttggccgcagtgttatcactcatggttatggcagcactgcataattctcttactgtcatgccatccgtaagatgcttttctgtgactggtgagtactcaaccaagtcattctgagaatagtgtatgcggcgaccgagttgctcttgcccggcgtcaatacgggataataccgcgccacatagcagaactttaaaagtgctcatcattggaaaacgttcttcggggcgaaaactctcaaggatcttaccgctgttgagatccagttcgatgtaacccactcgtgcacccaactgatcttcagcatcttttactttcaccagcgtttctgggtgagcaaaaacaggaaggcaaaatgccgcaaaaaagggaataagggcgacacggaaatgttgaatactcatactcttcctttttcaatattattgaagcatttatcagggttattgtctcatgagcggatacatatttgaatgtatttagaaaaataaacaaataggggttccgcgcacatttccccgaaaagtgccacctgacgtc
FLVCR2 (wild-type)	gacggatcgggagatctcccgatcccctatggtgcactctcagtacaatctgctctgatgccgcatagttaagccagtatctgctccctgcttgtgtgttggaggtcgctgagtagtgcgcgagcaaaatttaagctacaacaaggcaaggcttgaccgacaattgcatgaagaatctgcttagggttaggcgttttgcgctgcttcgcgatgtacgggccagatatacgcgttgacattgattattgactagttattaatagtaatcaattacggggtcattagttcatagcccatatatggagttccgcgttacataacttacggtaaatggcccgcctggctgaccgcccaacgacccccgcccattgacgtcaataatgacgtatgttcccatagtaacgccaatagggactttccattgacgtcaatgggtggagtatttacggtaaactgcccacttggcagtacatcaagtgtatcatatgccaagtacgccccctattgacgtcaatgacggtaaatggcccgcctggcattatgcccagtacatgaccttatgggactttcctacttggcagtacatctacgtattagtcatcgctattaccatggtgatgcggttttggcagtacatcaatgggcgtggatagcggtttgactcacggggatttccaagtctccaccccattgacgtcaatgggagtttgttttggcaccaaaatcaacgggactttccaaaatgtcgtaacaactccgccccattgacgcaaatgggcggtaggcgtgtacggtgggaggtctatataagcagagctctccctatcagtgatagagatctccctatcagtgatagagatcgtcgacgagctcgtttagtgaaccgtcagatcgcctggagacgccatccacgctgttttgacctccatagaagacaccgggaccgatccagcctccggactctagcgtttaaacttaagcttgccaccatggtgaatgaaggtcccaaccaggaagagagcgatgacacccctgtgccggagtccgcactccaagcggaccccagcgtctcggtccatcccagcgtctcggtccatcccagcgtctccatcaaccccagcgtctctgtccaccccagcagttcggcccaccccagtgccttagcccaacccagtggcttggctcaccccagtagctcgggccctgaggacctcagcgtgatcaaggtgagcaggcgccgttgggccgtggtcctggtgtttagctgctactccatgtgcaactcctttcagtggatccagtacggctccatcaataacatcttcatgcacttctacggtgtcagtgcctttgccattgactggctgtccatgtgctacatgctgacttacatccctctgctcctgccagtggcttggctgctggagaagttcggcctgcgcaccattgctctcactggctcggctctcaactgcctgggggcctgggtgaagctgggcagcctgaagccgcatctctttccggtcaccgtggtgggccagctcatctgctctgtggcccaggttttcatcctgggcatgccctcccgcatcgcttccgtctggttcggggctaatgaggtttcaacagcctgctccgtggctgtctttggcaatcagcttggaattgcgattgggttcttggtccctcctgttttggtacccaacattgaagaccgggacgagcttgcctaccacatcagcatcatgttctatataataggaggtgtggccactctcctcctcatccttgtcatcattgtgttcaaggagaaacctaaatatcccccagcagggcccaatccctgagctatgccttgacctctcctgatgcctcatacttaggttccatcgcccggctcttcaaaaatctcaactttgtgctgcttgtcatcacctatggtctgaatgctggtgctttttatgccttgtccactcttctgaatcgcatggtgatctggcactacccgggggaagaagtgaatgctggaagaattggcctgacgatcgtcattgcaggaatgcttggggctgtgatctcaggaatctggctggataggtccaaaacctacaaagagacaaccctggtagtctatatcatgacactggtgggcatggtggtgtacacgtttaccttgaacctgggacacctgtgggtagtgttcatcactgctggcacaatggcttctttatgactggctatctcccactgggatttgagtttgctgtggagctcacgtacccagaatcagaaggcatctcctccggcctcctcaacatatctgcacaggtatttgggatcatctttaccatctcccagggccagattattgacaactatggaaccaagcctgggaacatcttcctgtgtgtgttccttactcttggagcagccctcactgcattcattaaggcagatctccggagacagaaagcaaacaaagaaactcttgagaacaaactccaagaggaggaggaggagagcaacaccagcaaagtgcccactgctgtgtcagaggatcatctcgattacaaggatgacgacgataagtgagcggccgctcgagtctagagggcccgtttaaacccgctgatcagcctcgactgtgccttctagttgccagccatctgttgtttgcccctcc

	cccgtgccttccttgaccctggaaggtgccactcccactgtcctttcctaataaaatgaggaaattgcatcgcattgtctgagtaggtgtcattctattctggggggtggggtggggcaggacagcaa<br>gggggaggattgggaagacaatagcaggcatgctggggatgcggtgggctctatggcttctgaggcggaaagaaccagctggggctctagggggtatccccacgcgccctgtagcggcgcatta<br>agcgcggcgggtgtggtggttacgcgcagcgtgaccgctacacttgccagcgccctagcgcccgctcctttcgctttcttcccttcctttctcgccacgttcgccggctttccccgtcaagctctaaatc<br>gggggctccctttagggttccgatttagtgctttacggcacctcgaccccaaaaaacttgattagggtgatggttcacgtacctagaagttcctattccgaagttcctattctctagaaagtataggaa<br>cttccttggccaaaaagcctgaactcaccgcgacgtctgtcgagaagtttctgatcgaaaagttcgacagcgtctccgacctgatgcagctctcggagggcgaagaatctcgtgctttcagcttcga<br>tgtaggagggcgtggatatgtcctgcgggtaaatagctgcgccgatggtttctacaaagatcgttatgtttatcggcactttgcatcggccgcgctcccgattccggaagtgcttgacattggggaat<br>tcagcgagagcctgacctattgcatctcccgccgtgcacagggtgtcacgttgcaagacctgcctgaaaccgaactgcccgctgttctgcagccggtcgcggaggccatggatgcgatcgctgcgg<br>ccgatcttagccagacgagcgggttcggcccattcggaccgcaaggaatcggtcaatacactacatggcgtgatttcatatgcgcgattgctgatccccatgtgtatcactggcaaactgtgatgga<br>cgacaccgtcagtgcgtccgtcgcgcaggctctcgatgagctgatgctttgggccgaggactgccccgaagtccggcacctcgtgcacgcggatttcggctccaacaatgtcctgacggacaatgg<br>ccgcataacagcggtcattgactggagcgaggcgatgttcggggattcccaatacgaggtcgccaacatcttcttctggaggccgtggttggcttgtatggagcagcagacgcgctacttcgagcg<br>gaggcatccggagcttgcaggatcgccgcggctccgggcgtatatgctccgcattggtcttgaccaactctatcagagcttggttgacggcaatttcgatgatgcagcttgggcgcagggtcgatgc<br>gacgcaatcgtccgatccggagccgggactgtcgggcgtacacaaatcgcccgcagaagcgcggccgtctggaccgatggctgtgtagaagtactcgccgatagtggaaaccgacgccccagca<br>ctcgtccgagggcaaaggaatagcacgtactacgagatttcgattccaccgccgccttctatgaaaggttgggcttcggaatcgttttccgggacgccggctggatgatcctccagcgcggggatct<br>catgctggagttcttcgcccaccccaacttgtttattgcagcttataatggttacaaataaagcaatagcatcacaaatttcacaaataaagcatttttttcactgcattctagttgtggtttgtccaaa<br>ctcatcaatgtatcttatcatgtctgtataccgtcgacctctagctagagcttggcgtaatcatggtcatagctgtttcctgtgtgaaattgttatccgctcacaattccacacaacatacgagccggaa<br>gcataaagtgtaaagcctggggtgcctaatgagtgagctaactcacattaattgcgttgcgctcactgcccgctttccagtcgggaaacctgtcgtgccagctgcattaatgaatcggccaacgcgc<br>ggggagaggcggtttgcgtattgggcgctcttccgcttcctcgctcactgactcgctgcgctcggtcgttcggctgcggcgagcggtatcagctcactcaaaggcggtaatacggttatccacagaa<br>tcaggggataacgcaggaaagaacatgtgagcaaaaggccagcaaaaggccaggaaccgtaaaaaggccgcgttgctggcgtttttccataggctccgcccccctgacgagcatcacaaaaat<br>cgacgctcaagtcagaggtggcgaaacccgacaggactataaagataccaggcgtttccccctggaagctccctcgtgcgctctcctgttccgaccctgccgcttaccggatacctgtccgcctttct<br>cccttcgggaagcgtggcgctttctcatagctcacgctgtaggtatctcagttcggtgtaggtcgttcgctccaagctgggctgtgtgcacgaaccccccgttcagcccgaccgctgcgccttatccgg<br>taactatcgtcttgagtccaacccggtaagacacgacttatcgccactggcagcagccactggtaacaggattagcagagcgaggtatgtaggcggtgctacagagttcttgaagtggtggcctaa<br>ctacggctacactagaagaacagtatttggtatctgcgctctgctgaagccagttaccttcggaaaaagagttggtagctcttgatccggcaaacaaaccaccgctggtagcggtggtttttttgttt<br>gcaagcagcagattacgcgcagaaaaaaaggatctcaagaagatcctttgatcttttctacggggtctgacgctcagtggaacgaaaactcacgttaagggattttggtcatgagattatcaaaa<br>aggatcttcacctagatccttttaaattaaaaatgaagttttaaatcaatctaaagtatatatgagtaaacttggtctgacagttaccaatgcttaatcagtgaggcacctatctcagcgatctgtcta<br>tttcgttcatccatagttgcctgactccccgtcgtgtagataactacgatacgggagggcttaccatctggccccagtgctgcaatgataccgcgagacccacgctcaccggctccagatttatcagc<br>aataaaccagccagccggaagggccgagcgcagaagtggtcctgcaactttatccgcctccatccagtctattaattgttgccgggaagctagagtaagtagttcgccagttaatagtttgcgcaa<br>cgttgttgccattgctacaggcatcgtggtgtcacgctcgtcgtttggtatggcttcattcagctccggttcccaacgatcaaggcgagttacatgatcccccatgttgtgcaaaaaagcggttagctc<br>cttcggtcctccgatcgttgtcagaagtaagttggccgcagtgttatcactcatggttatggcagcactgcataattctcttactgtcatgccatccgtaagatgcttttctgtgactggtgagtactca<br>accaagtcattctgagaatagtgtatgcggcgaccgagttgctcttgcccggcgtcaatacgggataataccgcgccacatagcagaactttaaaagtgctcatcattggaaaacgttcttcgggg<br>cgaaaactctcaaggatcttaccgctgttgagatccagttcgatgtaacccactcgtgcacccaactgatcttcagcatcttttactttcaccagcgtttctgggtgagcaaaaacaggaaggcaaa<br>atgccgcaaaaaagggaataagggcgacacggaaatgttgaatactcatactcttcctttttcaatattattgaagcatttatcagggttattgtctcatgagcggatacatatttgaatgtatttaga<br>aaaataaacaaataggggttccgcgcacatttccccgaaaagtgccacctgacgtc

Supplementary Table 2: Amino acid sequences of human FLVCR variants used in this study.

Variant	Amino acid sequence
FLVCR1a (wild-type)	MARPDDEEGAAVAPGHPLAKGYLPLPRGAPVGKESVELQNGPKAGTFPVNGAPRDSLAAASGVLGGPQTPLAPEEETQARLLPAGAGAETPGAESSPLPL TALSPRRFVVLLIFSLYSLVNAFQWIQYSIISNVFEGFYGVTLLHIDWLSMVYMLAYVPLIFPATWLLDTRGLRLTALLGSGLNCLGAWIKCGSVQQHLFWVT MLGQCLCSVAQVFILGLPSRIASVWFGPKEVSTACATAVLGNQLGTAVGFLLPPVLVPNTQNDTNLLACNISTMFYGTSAVATLLFILTAIAFKEKPRYPPSQ AQAALQDSPPEEYSYKKSIRNLFKNIPFVLLLITYGIMTGAFYSVSTLLNQMILTYYEGEEVNAGRIGLTLVVAGMVGSILCGLWLDYTKTYKQTTLIVYILSFIG MVIFTFTLDLRYIIIVFVTGGVLGFFMTGYLPLGFEFAVEITYPESEGTSSGLLNASAQIFGILFTLAQGKLTSDYGPKAGNIFLCVWMFIGIILTALIKSDLRRHNI NIGITNVDVKAIPADSPTDQEPKTVMLSKQSESAIDYKDDDDK
FLVCFR2 (wild-type)	MVNEGPNQEESDDTPVPESALQADPSVSVHPSVSVHPSVSINPSVSVHPSSSAHPSALAQPSGLAHPSSSGPEDLSVIKVSRRRWAVVLVFSCYSMCNSFQ WIQYGSINNIFMHFYGVSAFAIDWLSMCYMLTYIPLLLPVAWLLEKFGLRTIALTGSALNCLGAWVKLGSLKPHLFPVTVVGQLICSVAQVFILGMPSRIASV WFGANEVSTACSVAVFGNQLGIAIGFLVPPVLVPNIEDRDELAYHISIMFYIIGGVATLLLILVIIVFKEKPKYPPSRAQSLSYALTSPDASYLGSIARLFKNLNFVL LVITYGLNAGAFYALSTLLNRMVIWHYPGEEVNAGRIGLTIVIAGMLGAVISGIWLDRSKTYKETTLVVYIMTLVGMVVYTFTLNLGHLWVVFITAGTMGFF MTGYLPLGFEFAVELTYPESEGISSGLLNISAQVFGIIFTISQGQIIDNYGTKPGNIFLCVFLTLGAALTAFIKADLRRQKANKETLENKLQEEEEESNTSKVPTAV SEDHLDYKDDDDK

Supplementary Table 3: Description of the different simulations performed in this study.

Protein	Orientation	Ligand	Box size [nm]	Duration [ns]	Release time [ns]	# atoms	# water molecules
FLVCR1	Inward-facing	Choline in cavity	10 x 10 x 11	1000	n.r.*	108536	21861
FLVCR1	Inward-facing	Choline in cavity	10 x 10 x 11	1000	n.r.	108536	21861
FLVCR1	Inward-facing	Choline in cavity	10 x 10 x 11	1000	n.r.	108536	21861
FLVCR1^{W125A}	Inward-facing	Choline in cavity	10 x 10 x 11	200	129	108522	21861
FLVCR1^{W125A}	Inward-facing	Choline in cavity	10 x 10 x 11	200	44	108522	21861
FLVCR1^{W125A}	Inward-facing	Choline in cavity	10 x 10 x 11	200	54	108522	21861
FLVCR1	Inward-facing	*As isolated*	10 x 10 x 11	1000	n.a.**	113323	23636
FLVCR1	Inward-facing	*As isolated*	10 x 10 x 11	1000	n.a.	113323	23636
FLVCR1	Inward-facing	*As isolated*	10 x 10 x 11	1000	n.a.	113323	23636
FLVCR1	Inward-facing	Protonated ethanolamine in cavity	10 x 10 x 11	430	224	107820	21793
FLVCR1	Inward-facing	Protonated ethanolamine in cavity	10 x 10 x 11	443	14	107820	21793
FLVCR1	Inward-facing	Protonated ethanolamine in cavity	10 x 10 x 11	467	142	107820	21793
FLVCR1	Inward-facing	Deprotonated ethanolamine in cavity	10 x 10 x 11	212	30	108525	21861
FLVCR1	Inward-facing	Deprotonated ethanolamine in cavity	10 x 10 x 11	615	560	108525	21861
FLVCR1	Inward-facing	Deprotonated ethanolamine in cavity	10 x 10 x 11	1000	910	108525	21861
FLVCR2	Outward-facing	Choline in solution [380mM]	10 x 10 x 13	1000	n.a.	133935	28101
FLVCR2	Outward-facing	Choline in solution [380mM]	10 x 10 x 13	1000	n.a.	133935	28101
FLVCR2	Outward-facing	Choline in solution [380mM]	10 x 10 x 13	1000	n.a.	133935	28101
FLVCR2	Inward-facing	Choline in cavity	10 x 10 x 11	1000	895	111807	23081
FLVCR2	Inward-facing	Choline in cavity	10 x 10 x 11	1000	n.r.	111807	23081
FLVCR2	Inward-facing	Choline in cavity	10 x 10 x 11	1000	n.r.	111807	23081
FLVCR2^{W102A}	Inward-facing	Choline in cavity	10 x 10 x 11	187	29	111793	23081
FLVCR2^{W102A}	Inward-facing	Choline in cavity	10 x 10 x 11	186	37	111793	23081
FLVCR2^{W102A}	Inward-facing	Choline in cavity	10 x 10 x 11	195	45	111793	23081
FLVCR2	Inward-facing	*As isolated*	10 x 10 x 11	1000	n.a.	113466	23640
FLVCR2	Inward-facing	*As isolated*	10 x 10 x 11	1000	n.a.	113466	23640
FLVCR2	Inward-facing	*As isolated*	10 x 10 x 11	1000	n.a.	113466	23640
FLVCR2	Outward-facing	*As isolated*	10 x 10 x 11	1000	n.a.	110751	22575
FLVCR2	Outward-facing	*As isolated*	10 x 10 x 11	1000	n.a.	110751	22575
FLVCR2	Outward-facing	*As isolated*	10 x 10 x 11	1000	n.a.	110751	22575

*n.a.: not applicable

**n.r.: no release observed

Supplementary Table 4: MD simulation checklist.

**Reliability and reproducibility checklist for molecular dynamics simulations**<br>***All boxes must be marked YES by acceptance unless "Response not needed if No".**	**Yes**	**No**	**Response**<br>**(Please state where this information can be found in the text)**
**1. Convergence of simulations and analysis**			
1a. Is an evaluation presented in the text to show that the property being measured has equilibrated in the simulations<br>(*e.g.* time-course analysis)?	☒	☐	Plots of distances between the ligands and residues in the cavity as a function of time demonstrate equilibration (Figs. 3c,d, 4b, and Supplementary Fig. 11).
1b. Then, is it described in the text how simulations are split into equilibration and production runs and how much data were analyzed from production runs?	☒	☐	Described in the Methods section.
1c. Are there at least 3 simulations per simulation condition with statistical analysis?	☒	☐	3 replicas were run for each system, see Methods.
1d. Is evidence provided in the text that the simulation results presented are independent of initial configuration?	☒	☐	Different replicas were started with randomized initial velocities (Methods). Extended Data Fig. 8 and Supplementary Fig. 12 show that ethanolamine can adopt different orientations in the binding cavity, independent from its initial position.
**2. Connection to experiments**			
2a. Are calculations provided that can connect to experiments (*e.g.* loss or gain in function from mutagenesis, binding assays, NMR chemical shifts, J-couplings, SAXS curves, interaction distances or FRET distances, structure factors, diffusion coefficients, bulk modulus and other mechanical properties, *etc.*)?	☒	☐	Simulations reflect the more pronounced dynamics of ethanolamine in the binding cavity as opposed to choline, which is in good agreement with the observed ligand densities from cryo-EM (Fig. 4d, Extended Data Fig. 8, and Supplementary Fig. 12). Figs. 3f,g, as well as Extended Data Fig. 7, connect the MD simulations to loss of choline transport activity upon mutagenesis.<br>Supplementary Fig. 6 confirms that distances between key residues at the internal and external gates observed with cryo-EM in different systems are maintained in MD simulations.
**3. Method choice**			
3a. Do simulations contain membranes, membrane proteins, intrinsically disordered proteins, glycans, nucleic acids, polymers, or cryptic ligand binding?	☒	☐	Simulations contain a POPC:POPG bilayer and membrane-embeded proteins FLVCR1 and FLVCR2.
3b. Is it described in the text whether the accuracy of the chosen model(s) is sufficient to address the question(s) under investigation (e.g. all-atom vs. coarse-grained models, fixed charge vs. polarizable force fields, implicit vs. explicit solvent or membrane, force field and water model, etc.)?	☒	☐	In this study, we used detailed atomistic simulations with the standard force field charmm36m together with improved parameters for cation-π interactions.
3c. Is the timescale of the event(s) under investigation beyond the brute-force MD simulation timescale in this study that enhanced sampling methods are needed?	☐	☒	
If **YES**, are the parameters and convergence criteria for the enhanced sampling method clearly stated?	☐	☐	
If **NO,** is the evidence provided in the text?	☒	☐	In our simulations, which access timescales of microseconds, we observe ethanolamine reorientation in the binding pocket, choline entry from solution into the cavity (Supplementary Fig. 10), and choline and ethanolamine release from the binding pocket (Supplementary Fig. 11).
**4. Code and reproducibility**			
4a. Is a table provided describing the system setup that includes simulation box dimensions, total number of atoms, total number of water molecules, salt concentration, lipid composition (number of molecules and type)?	☒	☐	Supplementary Table 3<br>Salt-concentration and lipid composition remain constant across all systems and are hence not included in the table. This information is mentioned in the Methods instead.
4b. Is it described in the text what simulation and analysis software and which versions are used?	☒	☐	See Methods.
4c. Are other parameters for the system setup described in the text, such as protonation state, type of structural restraints if applied, nonbonded cutoff, thermostat and barostat, etc.?	☒	☐	See Methods.
4d. Are initial coordinate and simulation input files and a coordinate file of the final output provided as supplementary files or in a public repository?	☒	☐	https://doi.org/10.5281/zenodo.10952971
4e. Is there custom code or custom force field parameters?	☐	☒	
If **YES**, are they provided as supplementary files or in a public repository?	☐	☒	

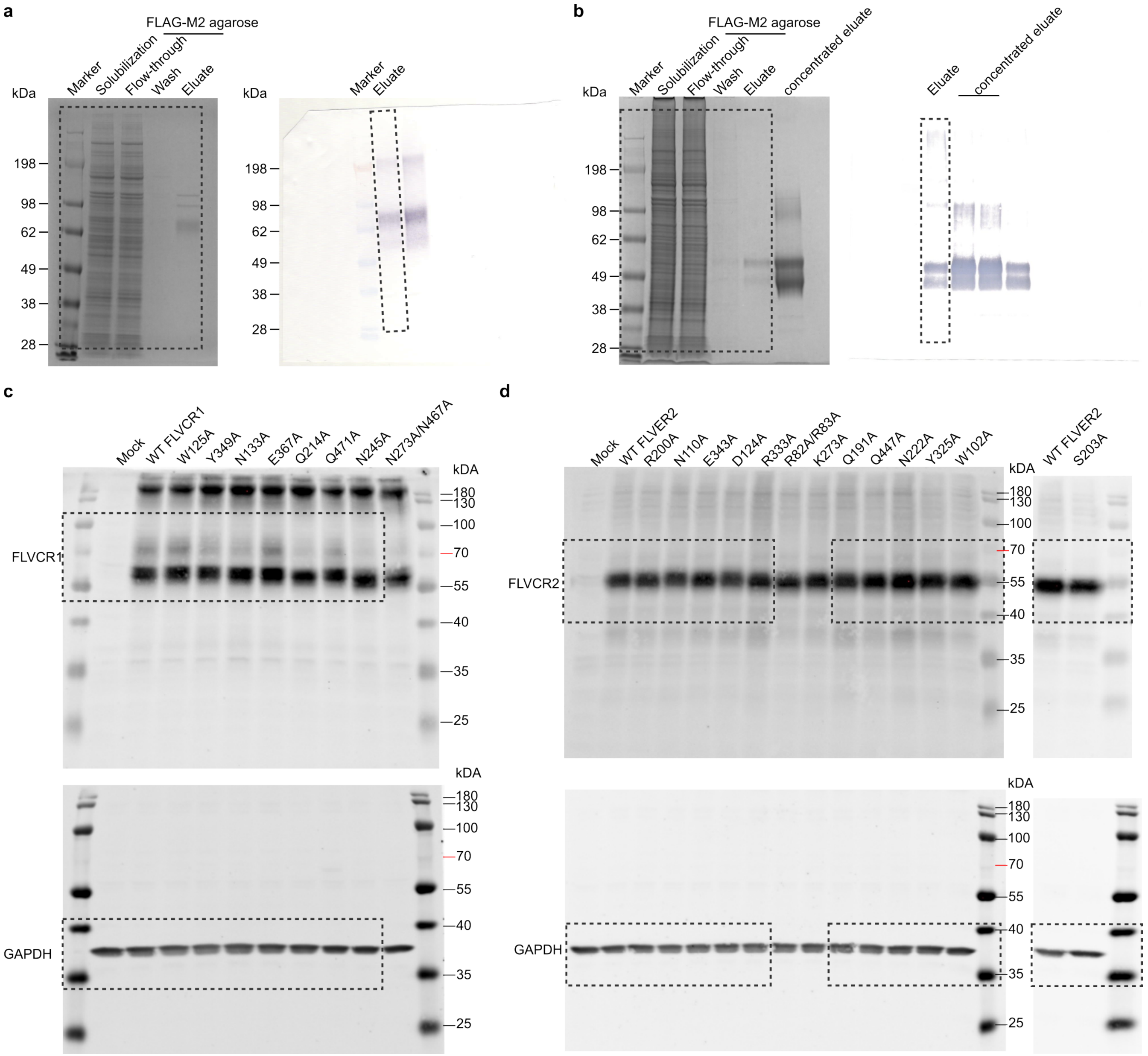

Supplementary Fig. 1: Uncropped gels and western blots. a, the uncropped SDS-PAGE gel (left) and western blot (right) of FLVCR1 purification shown in Supplementary Fig. 2e. **b**, the uncropped SDS-PAGE gel (left) and western blot (right) of FLVCR2 purification shown in Supplementary Fig. 2e. **c**, the uncropped western blots of FLVCR1 mutants shown in Supplementary Fig. 7c. **d**, the uncropped western blots of FLVCR2 mutants shown in Supplementary Fig. 7d. Dashed rectangles indicate the cropped images shown in the respective figures.

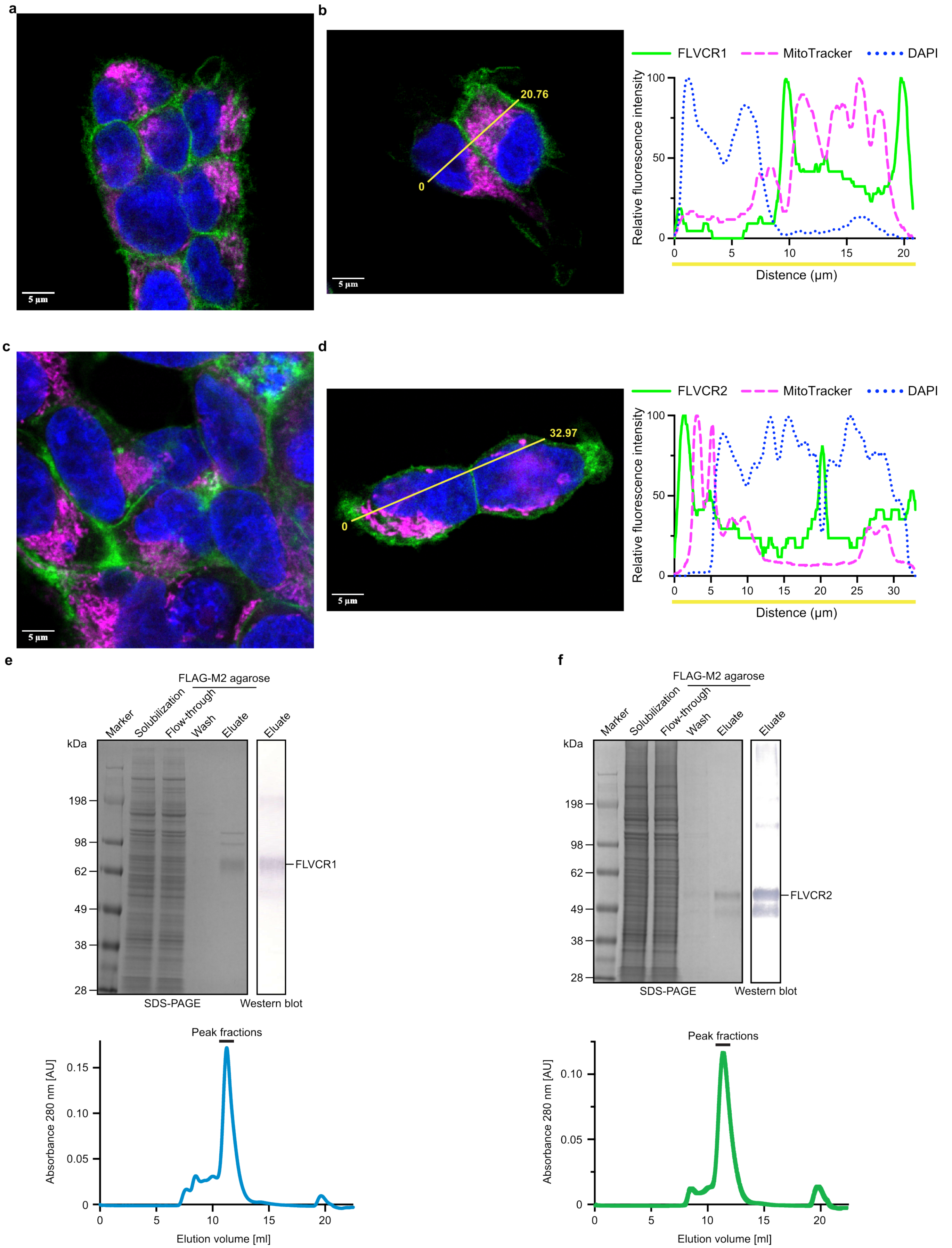

Supplementary Fig. 2: Purification of human FLVCR1 and FLVCR2. Representative immunofluorescence images of HEK293 cells overproducing FLAG-tagged wild-type human FLVCR1 (**a**,**b**) and FLVCR2 (**c**,**d**). Both FLVCRs were labelled with ANTI-FLAG® M2-FITC antibody. Mitochondria were labelled with MitoTracker™ Red CMXRos dyes. DNA was stained with DAPI. Distance trace of fluorescence intensities from these dyes within one representative image was analyzed and presented in (**b**) for FLVCR1 and (**d**) for FLVCR2. The experiment was performed three independent times, and multiple images at different magnification were collected from each sample. Representative images are shown. SDS-PAGE and western blot analysis of FLVCR1 (**e**) and FLVCR2 (**f**) affinity purification (top) and subsequent size-exclusion chromatography (bottom). The experiment was performed every time when overproduced proteins were purified. Representative images of gels and blots are shown. For gel source data, see Supplementary Fig. 1.

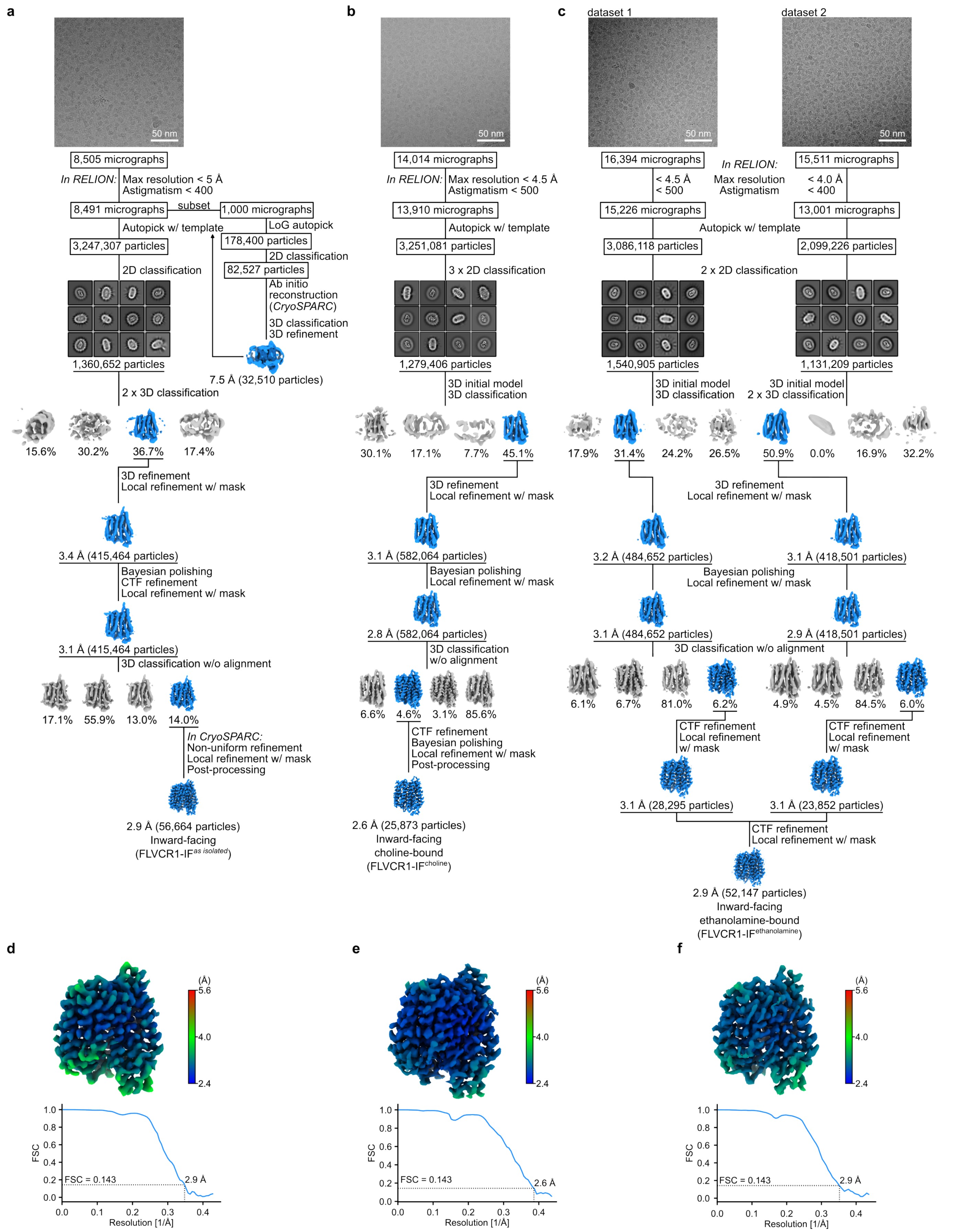

Supplementary Fig. 3: Single-particle cryo-EM analysis of human FLVCR1. a-c, Summary of the data processing procedure of the *as isolated* FLVCR1 sample and FLVCR1 supplemented with choline. **d-f**, Local resolution estimation (left) and Fourier shell correlation (FSC) curves (right) of the final cryo-EM maps of FLVCR1-IF$^{as\ isolated}$ and -IF^{choline}.

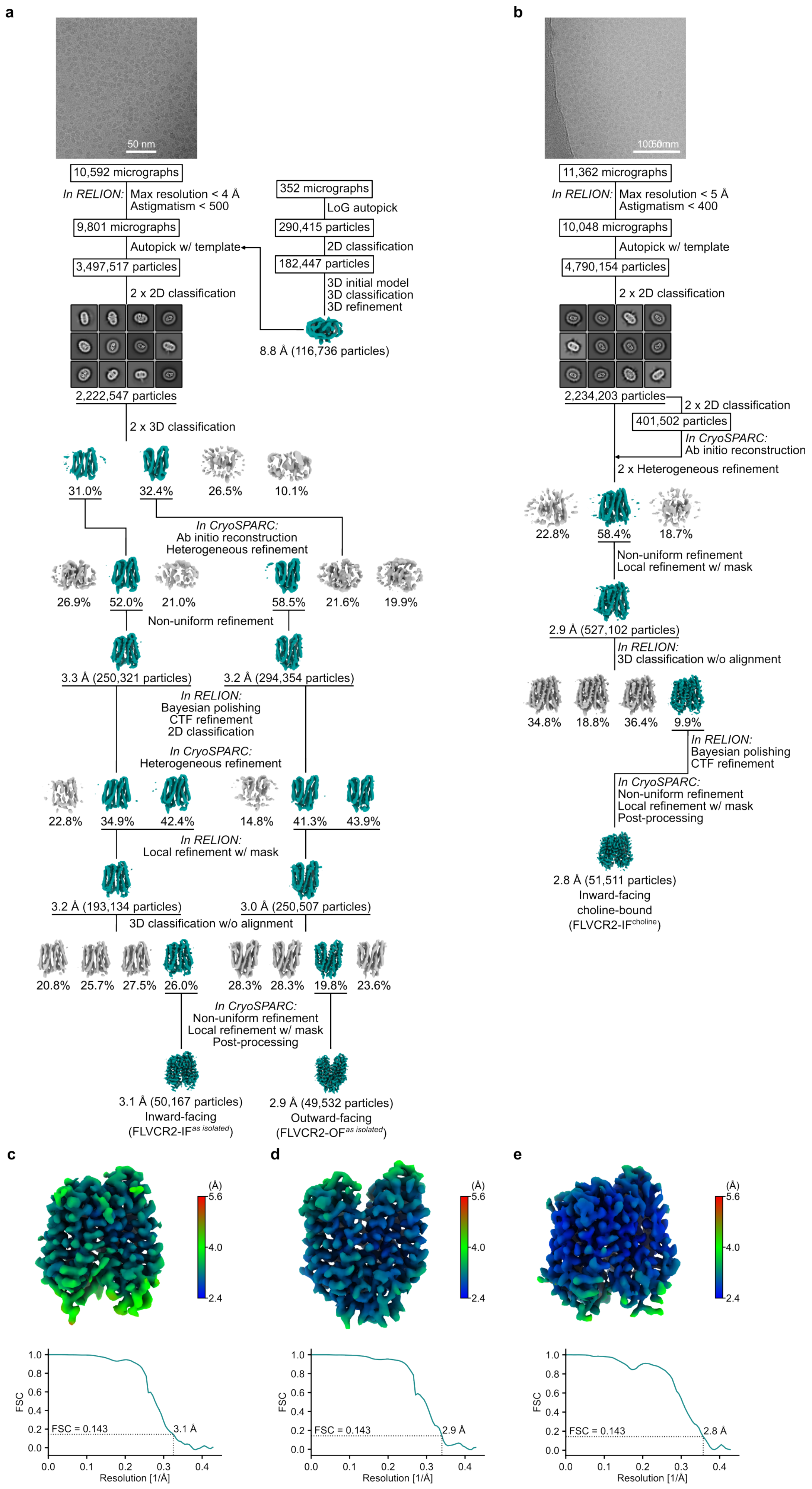
a
50 nm
10,592 micrographs
In RELION:
Max resolution < 4 Å
Astigmatism < 500
9,801 micrographs
Autopick w/ template
3,497,517 particles
2 x 2D classification
2,222,547 particles
2 x 3D classification
352 micrographs
LoG autopick
290,415 particles
2D classification
182,447 particles
3D initial model
3D classification
3D refinement
8.8 Å (116,736 particles)
31.0%
32.4%
26.5%
10.1%
In CryoSPARC:
Ab initio reconstruction
Heterogeneous refinement
26.9%
52.0%
21.0%
58.5%
21.6%
19.9%
Non-uniform refinement
3.3 Å (250,321 particles)
3.2 Å (294,354 particles)
In RELION:
Bayesian polishing
CTF refinement
2D classification
In CryoSPARC:
Heterogeneous refinement
22.8%
34.9%
42.4%
14.8%
41.3%
43.9%
In RELION:
Local refinement w/ mask
3.2 Å (193,134 particles)
3.0 Å (250,507 particles)
3D classification w/o alignment
20.8%
25.7%
27.5%
26.0%
28.3%
28.3%
19.8%
23.6%
In CryoSPARC:
Non-uniform refinement
Local refinement w/ mask
Post-processing
3.1 Å (50,167 particles)
Inward-facing
(FLVCR2-IF as isolated)
2.9 Å (49,532 particles)
Outward-facing
(FLVCR2-OF as isolated)
b
11,362 micrographs
In RELION:
Max resolution < 5 Å
Astigmatism < 400
10,048 micrographs
Autopick w/ template
4,790,154 particles
2 x 2D classification
2,234,203 particles
2 x 2D classification
401,502 particles
In CryoSPARC:
Ab initio reconstruction
2 x Heterogeneous refinement
22.8%
58.4%
18.7%
Non-uniform refinement
Local refinement w/ mask
2.9 Å (527,102 particles)
In RELION:
3D classification w/o alignment
34.8%
18.8%
36.4%
9.9%
In RELION:
Bayesian polishing
CTF refinement
In CryoSPARC:
Non-uniform refinement
Local refinement w/ mask
Post-processing
2.8 Å (51,511 particles)
Inward-facing
choline-bound
(FLVCR2-IF choline)
c
d
e
(Å)
5.6
4.0
2.4
FSC
FSC = 0.143
3.1 Å
2.9 Å
2.8 Å
Resolution [1/Å]
1.0
0.8
0.6
0.4
0.2
0.0
0.1
0.2
0.3
0.4

Supplementary Fig. 4: Single-particle cryo-EM analysis of human FLVCR2. **a**,**b**, Summary of the data processing procedure of the *as isolated* FLVCR2 and choline-supplemented FLVCR2 samples, respectively. **c**-**e**, Local resolution estimation (top) and FSC curves (bottom) of the final cryo-EM maps of FLVCR2-IF$^{\textit{as isolated}}$, FLVCR2-OF$^{\textit{as isolated}}$, and FLVCR2-IF^{choline}, respectively.

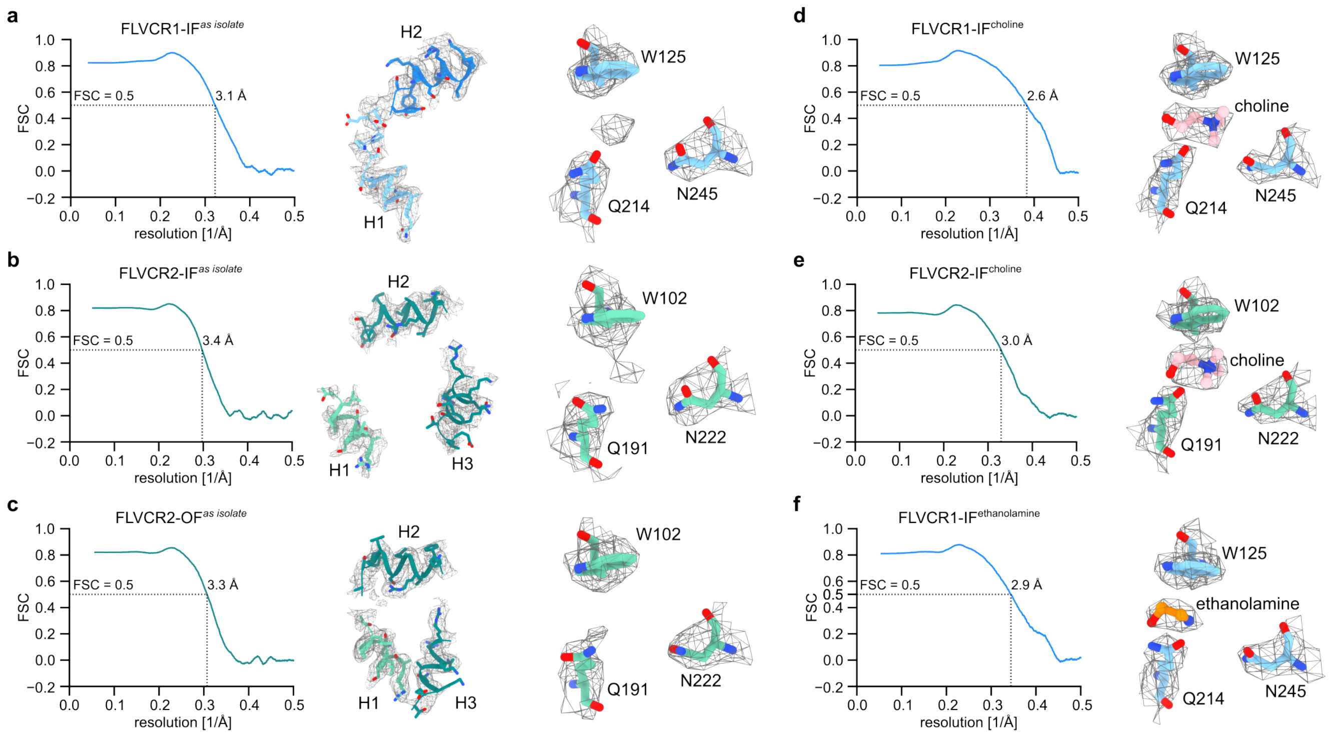

Supplementary Fig. 5: Cryo-EM density and model fitting. Cryo-EM map-to-model FSCs of FLVCR1-IF$^{as\ isolated}$ (**a**), FLVCR2-IF$^{as\ isolated}$ (**b**), FLVCR2-OF$^{as\ isolated}$ (**c**), FLVCR1-IF^{choline} (**d**), FLVCR2-IF^{choline} (**e**), and FLVCR1-IF^{ethanolamine} (**f**). The maps for the intracellular helices of the *as isolated* states as well as the maps for ligand-interacting residues are shown.

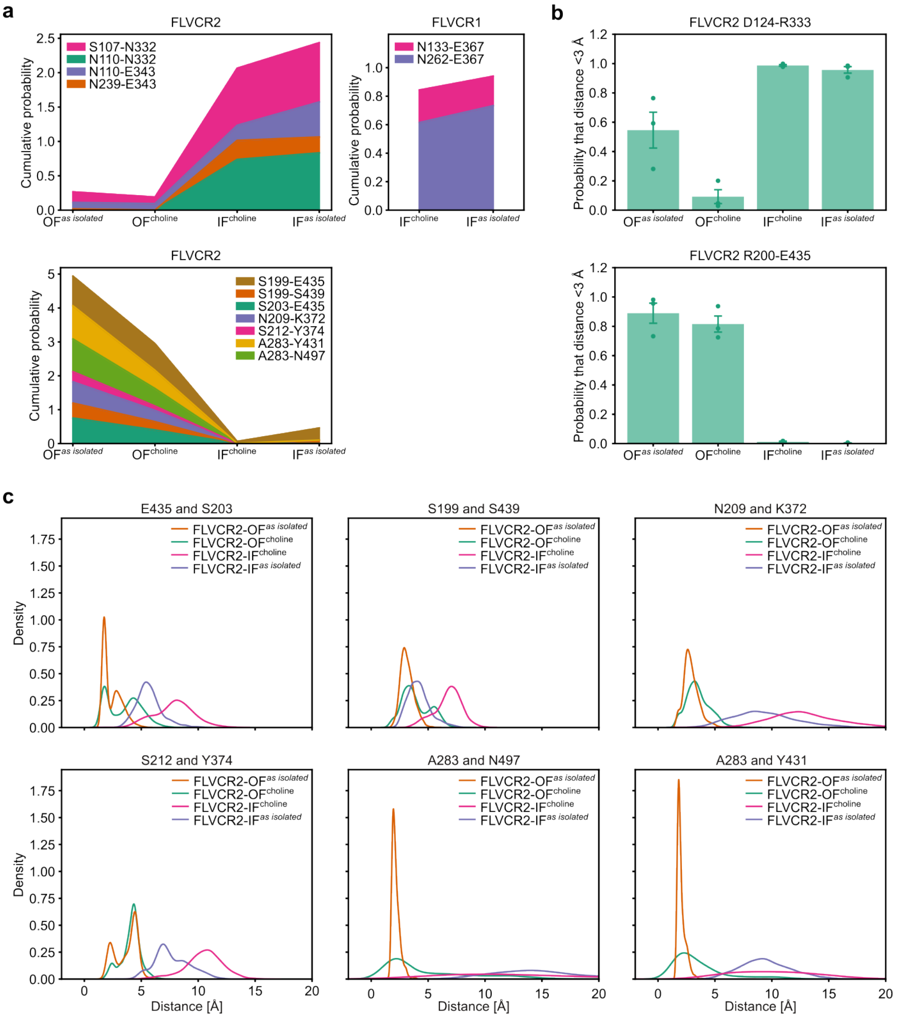

Supplementary Fig. 6: Persistence of critical interactions for stabilization of FLVCR1 and FLVCR2 in MD simulations. a, Cumulative probability of inter-domain hydrogen bond formation at the external gate (top) and the internal gate (bottom) with or without choline in the binding cavity. For each pair of residues, the probability was calculated as the fraction of the trajectory in which the distance between the residues remained below 3 Å. **b**, Stability of salt bridges within the external gate (top) and internal gate (bottom) of FLVCR2 in MD simulations. The displayed data represent the mean values obtained from three independent replicas, with error bars representing standard errors of the mean (s.e.m.). **c**, Distribution of distances between internal gate residues in MD simulations.

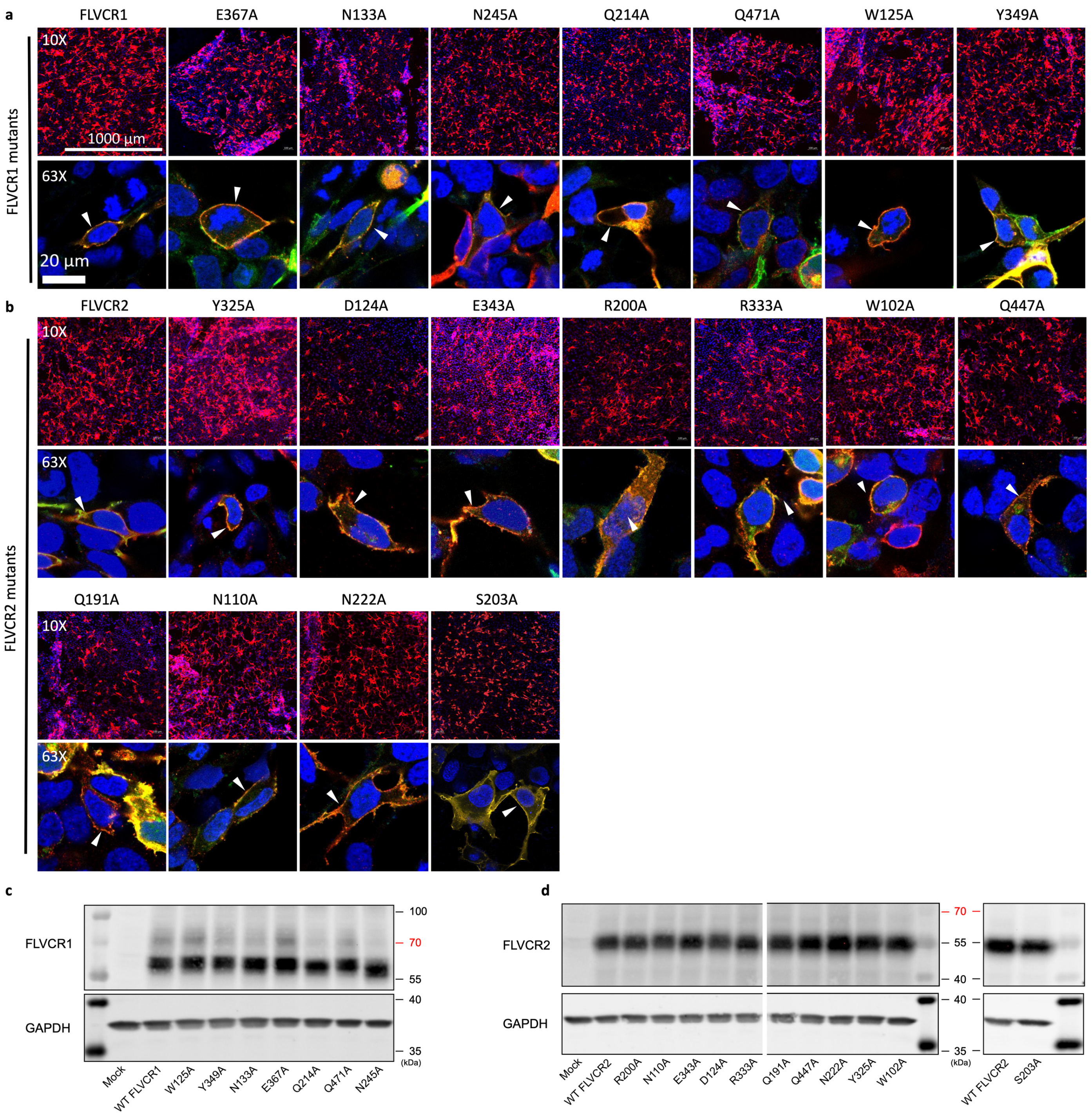

Supplementary Fig. 7: Immunofluorescent images and western blots of FLVCR mutants. Immunofluorescent images of cells overexpressing mutants of *FLVCR1* (**a**) or *FLVCR2* (**b**). Arrows indicate the localization of FLVCR variants. Scale bars represent 1000 µm for 10X, and 20 µm for 63X magnification. Experiment was performed once. Representative images are shown. Western blot analysis of mutant proteins from overexpression in HEK293 cell lysates of FLVCR1 (**c**) or FLVCR2 (**d**). GAPDH was used as a loading control. Protein samples were analyzed in two membranes with similar results. Representative images are shown. For gel source data, see Supplementary Fig. 1.

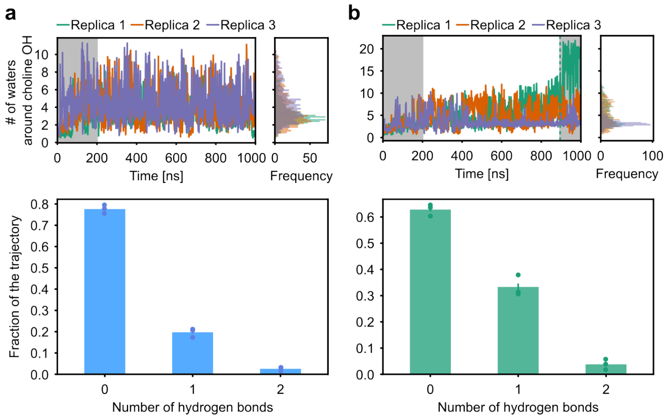

Supplementary Fig. 8: Hydration of choline in MD simulations. Interactions between choline and water in the binding site of inward-facing FLVCR1 (**a**) and FLVCR2 (**b**). The upper panels indicate the number of water molecules in the proximity of the hydroxyl group of choline with a cutoff of 5 Å in the $O^{choline}$-O^{water} distance. The first 200 ns of simulations were considered as equilibration and thus not included in the histograms. The last 105 ns of replica 1 in the FLVCR2 simulation were discarded as well due to choline release. The lower panels represent the hydrogen bond formation between water molecules and the hydroxyl group of choline in the simulations with a distance and angle cut-off of 3 Å and 20 °, respectively. Error bars represent s.e.m.

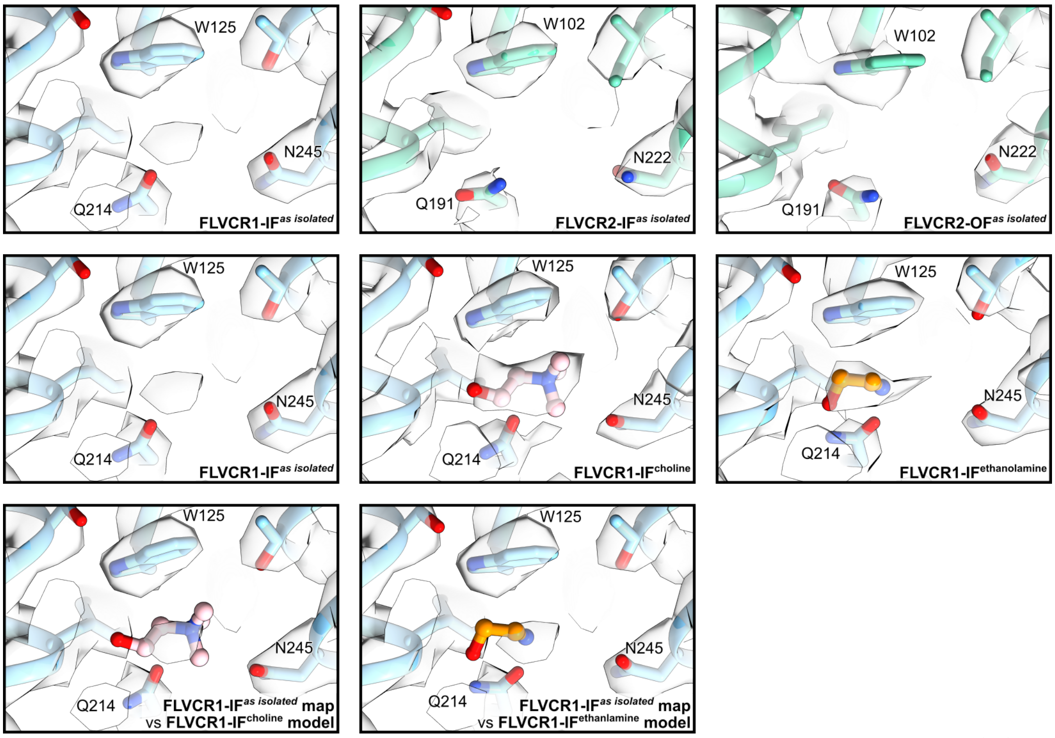

Supplementary Fig. 9: Cryo-EM density of the ligand-binding site in FLVCR1 and FLVCR2. Cryo-EM maps and models of FLVCR1-IF$^{as\ isolated}$, FLVCR1-IF^{choline}, FLVCR1-IF^{ethanolamine}, FLVCR2-IF$^{as\ isolated}$, and FLVCR2-OF$^{as\ isolated}$ states are shown in the upper two rows. Bottom panels are the superposition of the models of FLVCR1-IF^{choline} and FLVCR1-IF^{ethanolamine} into the cryo-EM map of FLVCR1-IF$^{as\ isolated}$. All maps are lowpass-filtered to 3.1 Å and adjusted to similar contour level.

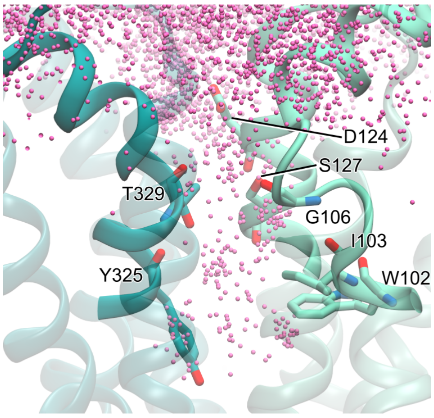

Supplementary Fig. 10: Choline entry from solution into the outward-facing cavity of FLVCR2 in MD simulations. Residues that interact with choline frequently are shown as sticks. Pink dots represent choline positions in all frames of one 1-μs trajectory.

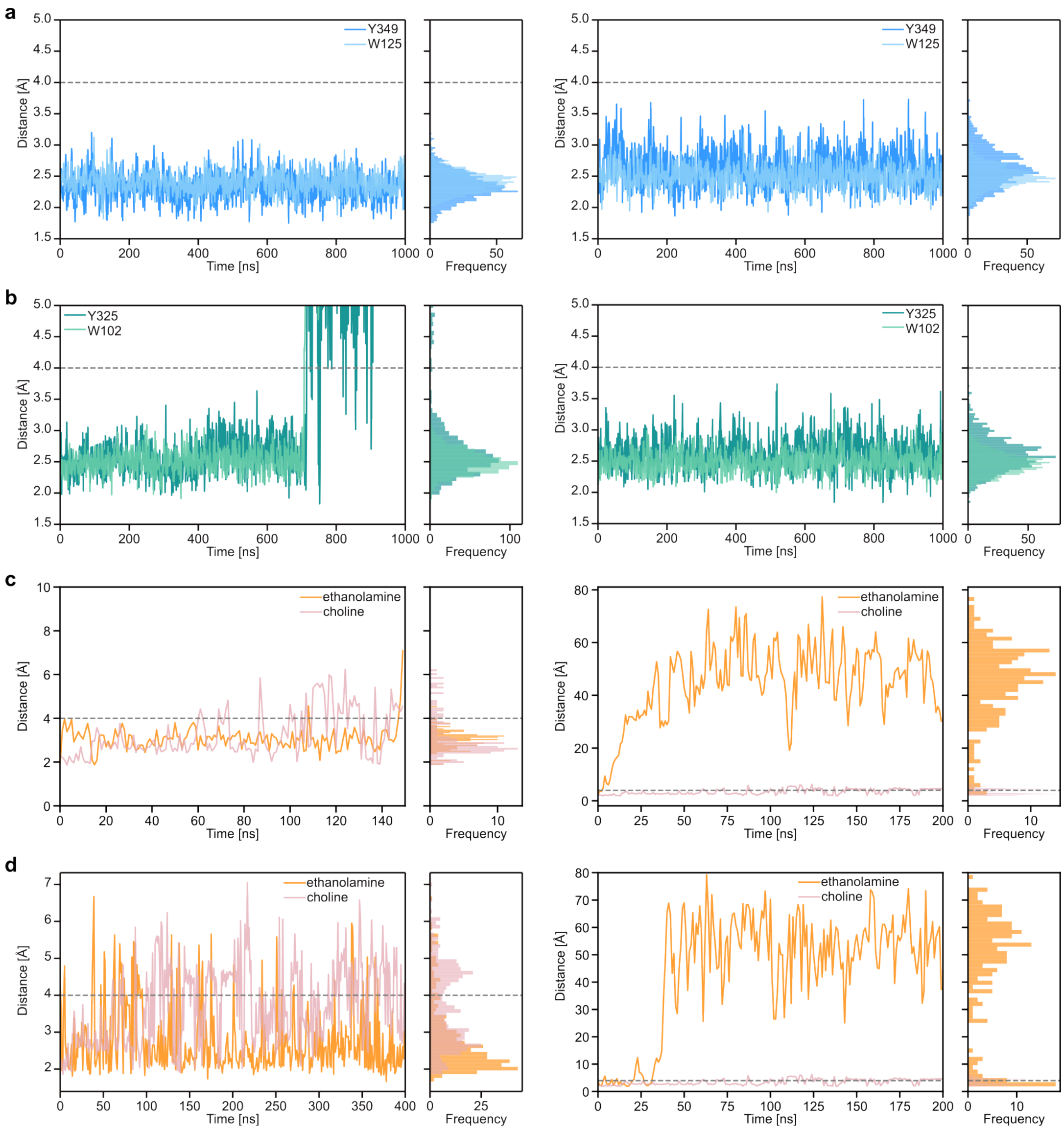

Supplementary Fig. 11: Distances between ligands and binding residues in different replicas. Time-resolved interactions of choline in inward-facing FLVCR1 (**a**) and FLVCR2 (**b**), and of protonated ethanolamine (**c**) and unprotonated ethanolamine (**d**) in inward-facing FLVCR1. In the first replica of (**b**), choline is released from FLVCR2 after ~750 ns. In the second replica of (**c**), ethanolamine leaves the binding pocket after only 5 ns. In the second replica of (**d**), ethanolamine leaves the binding pocket after only ~25 ns. In (**a**) and (**b**), for each frame, the minimum distance between the ligand and the side chain of the highly conserved W/Y residues was calculated. In (**c**) and (**d**), only the O atom of ethanolamine was included as described in Fig. 4.

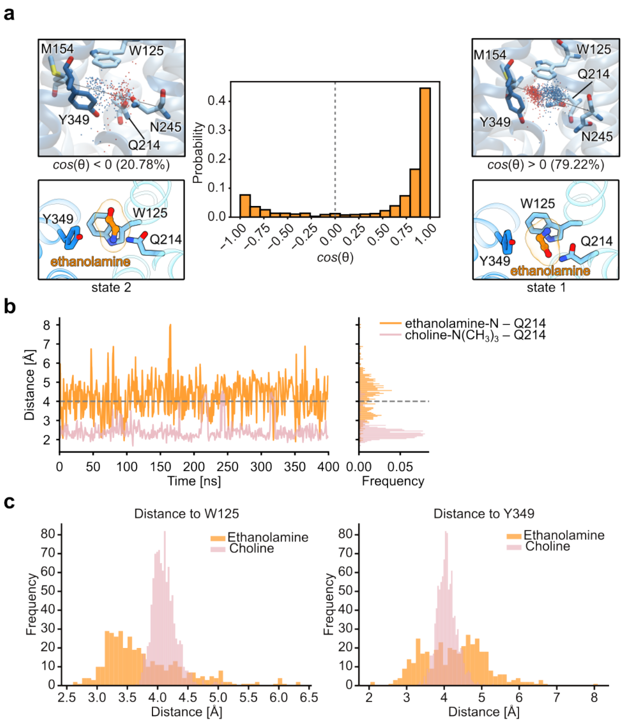

Supplementary Fig. 12: Interactions and dynamics of deprotonated ethanolamine binding to inward-facing FLVCR1. a, In MD simulations, deprotonated ethanolamine binds to inward-facing FLVCR1 in two distinct orientations, as quantified by the histogram of the cosine of the angle $\boldsymbol{\theta}$ between the vectors connecting the N and O atoms of ethanolamine, and the Cα atoms of N245^{FLVCR1} and M154^{FLVCR1} as described in Extended Data Fig. 9. The population with negative *cos*(θ) is higher than that in simulations with protonated ethanolamine (Extended Data Fig. 9a). **b**, Distance between Q214^{FLVCR1} and the primary amine of ethanolamine (orange) and, for reference, the tertiary amine of choline (pink) as function of time in MD simulations, with distance distributions on the right. **c**, Distribution of distances between the N-atom of ethanolamine or choline and the highly conserved tryptophan and tyrosine residues in MD simulations of FLVCR1.

Supplementary Video 1:
Structural transition of FLVCR2 from the outward-facing as isolated state to the inward-facing as isolated state. The model of FLVCR2 is shown in ribbon representation, with N-domain in light green and C-domain in dark green.

Supplementary Video 2:
Simulation of FLVCR2 in the inward-facing conformation with choline at the binding site. The ligand shows a stable interaction with W102 (blue) and transient contacts with water molecules, S99 (magenta), Q191 (yellow) and Q447 (red).

Supplementary Video 3:
Simulation of choline entry in the outward-facing cavity of FLVCR2 from solution. After 613 ns, choline transiently moves to a position below W102. Water and ions are omitted for clarity, phosphate groups of membrane lipids are shown as pink spheres.

Supplementary Video 4:
Choline release from the inward-facing cavity of FLVCR2. Water and ions are omitted for clarity. Phosphate groups of membrane lipids are shown as pink spheres. S199 and E435 are highlighted in magenta and red, respectively.
