# Supplementary material for: Molecular mechanism of choline and ethanolamine transport in humans
Source: Nature. 2024 May 22;630(8016):501–8. doi: 10.1038/s41586-024-07444-7 (PMC11168923; doi:10.1038/s41586-024-07444-7)
Supplement: Supplementary file 2 — Reporting Summary [file 41586_2024_7444_MOESM2_ESM.pdf]

nature portfolio

Corresponding author(s): Dr. Schara Safarian, Dr. Long N. Nguyen, Dr. Di Wu, Dr. Gerhard Hummer

Last updated by author(s): Apr 11, 2024

Reporting Summary

Nature Portfolio wishes to improve the reproducibility of the work that we publish. This form provides structure for consistency and transparency in reporting. For further information on Nature Portfolio policies, see our Editorial Policies and the Editorial Policy Checklist.

Statistics

For all statistical analyses, confirm that the following items are present in the figure legend, table legend, main text, or Methods section.

n/a	Confirmed	
☐	☒	The exact sample size (n) for each experimental group/condition, given as a discrete number and unit of measurement
☐	☒	A statement on whether measurements were taken from distinct samples or whether the same sample was measured repeatedly
☐	☒	The statistical test(s) used AND whether they are one- or two-sided<br>*Only common tests should be described solely by name; describe more complex techniques in the Methods section.*
☒	☐	A description of all covariates tested
☐	☒	A description of any assumptions or corrections, such as tests of normality and adjustment for multiple comparisons
☐	☒	A full description of the statistical parameters including central tendency (e.g. means) or other basic estimates (e.g. regression coefficient) AND variation (e.g. standard deviation) or associated estimates of uncertainty (e.g. confidence intervals)
☐	☒	For null hypothesis testing, the test statistic (e.g. F, t, r) with confidence intervals, effect sizes, degrees of freedom and P value noted<br>*Give P values as exact values whenever suitable.*
☐	☒	For Bayesian analysis, information on the choice of priors and Markov chain Monte Carlo settings
☒	☐	For hierarchical and complex designs, identification of the appropriate level for tests and full reporting of outcomes
☒	☐	Estimates of effect sizes (e.g. Cohen's d, Pearson's r), indicating how they were calculated

Our web collection on statistics for biologists contains articles on many of the points above.

Software and code

Policy information about availability of computer code

Data collection	The radioactivity assays were quantified by scintillation counter Tri-Carb (Perkin Elmer). The immunofluorescent staining samples were imaged with laser confocal microscopes (Zeiss LSM710 and Leica STELLARIS 5). Titan Krios microscopes operated at 300 kV and equipped with a Selectris X imaging filter and a Falcon4 camera (Thermo Fisher) or a BioQuantum energy filter and a K3 camera (Gatan); Data collection quality was monitored through EPU v. 3.0-3.4 and CryoSparc Live v3.0 and 4.0. Differential scanning fluorimetry of purified FLVCR variants were investigated with a Prometheus Panta (NanoTemper, no other version available). All molecular dynamics simulations were performed using the GROMACS v.2022.4 software. Biorender was used to draw cartoon and illustration.
Data analysis	For cryo-EM data analysis: ChimeraX v.1.5 and 1.6; MotionCor2-2.1.2.6; Gctf v.1.06; CLUSTAL Omega v.1.2.4; RELION-3.1 and -4.0; CryoSPARC Live v3.0 and v4.0; COOT v. 0.8.9; Phenix (v1.18); MolProbity v.4.5. For molecular dynamics simulation analysis: CHARMM36m force field was used for protein, lipids, heme and ions, together with TIP3P water; LINCS algorithm was used to constrain the bonds involving hydrogen atoms; Visual Molecular Dynamics (VMD) v.1.9.3 and GROMACS v.2022.4; PROPKA server v.3.5.0 was used to predicts the pKa values of ionizable groups; Pymol v.3.0 was used to introduce mutations; gmx_MMPBSA v1.6.2 was used for energy calculation. NanoDSF data was analyzed via PR.Panta Analysis v1.4.4 and Python libraries numpy v.1.21.5, scipy v.1.7.3, pandas v.1.5.2, matplotlib v.3.6.2, and seaborn v.0.12.2 in Microsoft Visual Studio Code v.1.80.1. Tunnels and cavities were mapped with MOLE 2.5, and calculated with CASTp. ConSurf server (https://consurf.tau.ac.il/) was used for conservation analysis. Online version of BioRender was used for illustration generation.

For manuscripts utilizing custom algorithms or software that are central to the research but not yet described in published literature, software must be made available to editors and reviewers. We strongly encourage code deposition in a community repository (e.g. GitHub). See the Nature Portfolio guidelines for submitting code & software for further information.

Data

Policy information about availability of data

All manuscripts must include a data availability statement. This statement should provide the following information, where applicable:

- Accession codes, unique identifiers, or web links for publicly available datasets
- A description of any restrictions on data availability
- For clinical datasets or third party data, please ensure that the statement adheres to our policy

Cryo-EM maps are deposited to the Electron Microscopy Data Bank under accession numbers: EMD-18334, EMD-18335, EMD-18336, EMD-18337, EMD-18339, EMD-19009. Atomic models of human FLVCR1 and FLVCR2 have been deposited to the Protein Data Bank under accession numbers: 8QCS, 8QCT, 8QCX, 8QCY, 8QD0, 8R8T. All molecular dynamics trajectories generated for this study and simulation input files are deposited in a Zenodo repository and freely available via the following DOI: 10.5281/zenodo.10952971. All other data is presented in the main text or supplementary materials. Source data are provided with this paper.

Research involving human participants, their data, or biological material

Policy information about studies with human participants or human data. See also policy information about sex, gender (identity/presentation), and sexual orientation and race, ethnicity and racism.

Reporting on sex and gender	N/A
Reporting on race, ethnicity, or other socially relevant groupings	N/A
Population characteristics	N/A
Recruitment	N/A
Ethics oversight	N/A

Note that full information on the approval of the study protocol must also be provided in the manuscript.

Field-specific reporting

Please select the one below that is the best fit for your research. If you are not sure, read the appropriate sections before making your selection.

☒ Life sciences ☐ Behavioural & social sciences ☐ Ecological, evolutionary & environmental sciences

For a reference copy of the document with all sections, see nature.com/documents/nr-reporting-summary-flat.pdf

Life sciences study design

All studies must disclose on these points even when the disclosure is negative.

Sample size	Sample sizes (number of collected micrographs) of respective cryo-EM datasets were chosen based on instrument availability and experimental design. Datasets of > 5000 micrographs ensured a sufficient number of particles to achieve resolutions < 3.5 Å. The smallest collected dataset contained 5356 micrographs (FLVCR2 supplemented with heme dataset) while the largest dataset contained 14014 micrographs (FLVCR1 supplemented with choline dataset). Nano differential scanning fluorimetry were performed in technical replicates (NanoDSF, n = 3). Technical replicates were chosen to determine standard deviation values for each data points and to validate data quality. For functional assays, no predetermined sample size was applied. For these experiments, 3 biological replicates were always used in each experiment. Most of the functional assays were repeated 2-3 times. However, only one dataset was shown in the manuscript.
Data exclusions	No data were excluded.
Replication	Single particle cryo-EM is based on averaging protein particles of nearly identical orientation within a vitreous layer of ice. Therefore, replication is not per se required to ensure statistical robustness of structural data. In case of this work, we have determined 2 individual structures of FLVCR1 and 4 individual structures of FLVCR2 under different sample conditions. Hence these data can be considered as biological replicates of the presented structural data.<br>For the functional assays (testing the activity of the mutants), we have performed the experiments with at least 3 biological replicates using different concentrations of choline and ethanolamine (the ligands). However, we chose to use the concentrations in which the datasets are used in the current manuscript. The results of transport assays using different concentrations of choline and ethanolamine are also consistent with the results used in the manuscript.<br>For the other functional assays, we repeated the experiments using the same conditions at least twice. These results are also included in the SOURCE data file, but please note that only one dataset was used to draw the graphs in the manuscript.
Randomization	Generally, no randomization was required for the experimental design of this study. However, it is to note that particles are randomized during data processing steps in Relion and cryoSPARC (randomization during 2D classification, randomized half sets during Refine3D). Randomized half sets of particles are used in final reconstruction steps in order to determined gold-standard Fourier shell correlations based

on the 0.143 level.
For the functional assays, Western blot analysis and IF of the mutants, we did not randomize the samples as the ID of the mutant plasmids used for transfection are known by the experimentalists.

Blinding

For all studies in this manuscript, such as 3D reconstruction, or NanoDSF measurements, there was no awareness of group assignment that caused biased results, so blinding was was relevant for data reliability.

Reporting for specific materials, systems and methods

We require information from authors about some types of materials, experimental systems and methods used in many studies. Here, indicate whether each material, system or method listed is relevant to your study. If you are not sure if a list item applies to your research, read the appropriate section before selecting a response.

Materials & experimental systems

n/a	Involved in the study
☐	☒ Antibodies
☐	☒ Eukaryotic cell lines
☒	☐ Palaeontology and archaeology
☐	☒ Animals and other organisms
☒	☐ Clinical data
☒	☐ Dual use research of concern
☒	☐ Plants

Methods

n/a	Involved in the study
☒	☐ ChIP-seq
☒	☐ Flow cytometry
☒	☐ MRI-based neuroimaging

Antibodies

Antibodies used

Monoclonal ANTI-FLAG® M2 antibody produced in mouse - clone M2, purified immunoglobulin (Purified IgG1 subclass), buffered aqueous solution (10 mM sodium phosphate, 150 mM NaCl, pH 7.4, containing 0.02% sodium azide), Merck (formly Sigma-Aldrich), F3165; Anti-Mouse IgG (whole molecule) - Alkaline Phosphatase antibody produced in goat, Merck (formly Sigma-Aldrich), A9316.
FLAG-tagged FLVCR1 and FLVCR2 were detected using anti-FLAG (F3165, Sigma-Aldrich) antibodies at 1:1,000 dilution. Anti-mouse IgG antibody conjugated with alkaline phosphatase (A9316, Sigma-Aldrich) was used as secondary antibody at 1:5,000 dilution.
Native FLVCR1 and FLVCR2 proteins were detected by polyclonal FLVCR1 and FLVCR2 antibodies raised in-house at 1:1,000 dilution.
GAPDH antibody (sc-32233, Santa Cruz) was used at 1:4,000 dilution. Monoclonal ANTI-FLAG® M2-FITC antibody produced in mouse (F4049, Sigma-Aldrich) was used at final concentration of 10 µg/mL in TBS for immunofluorescent staining against the FLAG-tags in the overproduction stable cells. Alexa Fluor 555 (A-21428, Invitrogen™, ThermoFisher, Lot 2527964, polyclonal Goat anti-Rabbit IgG) was used as secondary antibody at 1:500 dilutions for immunofluorescent staining against the polyclonal FLVCR1 and FLVCR2 primary antibodies for HEK293 cells.
Details of other antibodies used in the study was included in the manuscript as well.

Validation

Primary antibody Monoclonal ANTI-FLAG® M2 antibody produced in mouse was validated by the manufacturer:
Sensitivity Test - Detects 2 ng of FLAG-BAP fusion protein by dot blot using chemiluminescent detection.
Specificity - Detects a single band of protein on a western blot from an E. coli crude cell lysate.
For detailed information please see: https://www.sigmaaldrich.com/DE/en/product/sigma/f3165
Primary antibody Monoclonal ANTI-FLAG® M2-FITC antibody was validated by the manufacturer:
Immunoflourescence (Direct) performed using CMV-2 transfected COS-7 cells.
For detailed information please see: https://www.sigmaaldrich.com/DE/en/product/sigma/f4049
Primary antibody polyclonal FLVCR1 and FLVCR2 antibodies against human proteins were validated using cells with and without FLVCR1 and FLVCR2 genes.

Eukaryotic cell lines

Policy information about cell lines and Sex and Gender in Research

Cell line source(s)

Flp-In™ T-REx™ 293 Cell Line: cells are purchased from Thermo Fisher (formly Invitrogen™). HEK293 cells originally from ATCC (CRL-1573) were used.

Authentication

No further authentication was performed for the commercially available cell line.

Mycoplasma contamination

Periodically test negative.

Commonly misidentified lines
(See ICLAC register)

No commonly misidentified cell lines were used in this study.

Animals and other research organisms

Policy information about studies involving animals; ARRIVE guidelines recommended for reporting animal research, and Sex and Gender in Research

Laboratory animals	Mouse tissues were used in the study. These mice include FLVCR1f/f-Mx1Cre and control mice (FLVCR1f/+Mx1Cre and FLVCR1f/f). Tissues were collected from mice at 3-6 months old.
Wild animals	No wild animal was used in this study.
Reporting on sex	Gender of mice was not reported.
Field-collected samples	No field collected sample was used in this study.
Ethics oversight	Studies involving mice were reviewed and approved by the University of Washington Institutional Animal Care and Use Committee under protocol number 2001-13.

Note that full information on the approval of the study protocol must also be provided in the manuscript.
